# Coral larval proteomics at onset of metamorphosis highlights innate immunity maturation in parallel to neuro-sensing and skeletal development

**DOI:** 10.3389/fphys.2026.1763453

**Published:** 2026-02-19

**Authors:** Soheib Djamaa, Arul Marie, Alain Paris, Séverine Zirah, Isabelle Domart-Coulon

**Affiliations:** Molécules de Communication et Adaptation des Micro-organismes, UMR 7245CNRS / Muséum National d'Histoire Naturelle (MNHN), Paris, France

**Keywords:** coral, larval settlement, mass spectrometry, metamorphosis, Pocillopora acuta, proteomics

## Abstract

**Introduction:**

Coral larval metamorphosis is a critical life cycle transition from swimming planula to benthic polyp, key for reproductive success and survival of reef populations. The larval physiological processes involved during this transition are relatively unknown, in the absence of exogenous microbial induction.

**Methods:**

Here the identity and abundance of coral proteins detected at onset of metamorphosis (swimming planula to ‘settler’ stages) was investigated using planulae released from 4 distinct brooding Pocillopora acuta coral colonies, to consider maternal carry-over effects on the variability of larval proteomes.

**Results and discussion:**

NanoLC-MS/MS data analysis identified a total of 5,570 coral proteins, of which 1,119 occurred only either in planula or settler. Label-free quantification revealed 102 differentially enriched proteins (DEPs, log2 fold change > |2| p-value < 0.05), categorized into 7 predicted functional groups: adhesion and cytoskeleton remodeling, neurosensing, biocalcification toolkit, metabolism, morphogenesis, innate immunity, and antioxidant stress defense. Additionally, 98 aboral unique proteins and 25 aboral DEPs were quantified in the bisected planula, consistent with the presence of specialized aboral cell types involved in sensing of environmental cues. These results reveal the activation of coral innate immunity during larval metamorphosis, providing better knowledge of coral settlement physiology, with potential future ecological applications.

## Introduction

Coral larval metamorphosis is a critical life cycle transition from swimming planula to benthic polyp, key for reproductive success and the survival of reef populations (Gleason and Hofmann, 2011). Scleractinian, “stony”, corals have an indirect life cycle with two different modes of reproduction (Wallace, 1990; Gleason and Hofmann, 2011). Spawners release eggs and sperm (gametes) into the seawater where external fertilization takes place. Brooders undergo internal fertilization and embryo-to-larva development within the polyp tissue of the parent (mother) colony, and release swimming planula larvae, which are competent to settle. Planulae swim and drift in the water column until they sense inductive cues to settle, emitted by selected benthic microhabitat [reviewed in (Harrington et al., 2004; Ritson-Williams et al., 2016; Speare et al., 2023)]. Typical of probing behavior, the cylindrical-shaped planula contracts reversibly into a rotating disk, compressed along the oral/aboral axis, with aboral pole making repeated contacts with the available surfaces. Commitment to metamorphosis is morphologically characterized by flattened shape and major larval-to-polyp tissue remodeling, with onset of aboral calcification and oral tentacle budding. The settling post-larva attaches to the substrate then continues to grow into a primary polyp, with subsequent lateral budding of additional secondary polyps, forming a juvenile colony that will eventually mature into an adult colony. At adult stage, it will be capable of producing and releasing new planula larvae (planulation), thus completing its life cycle. Morphological criteria routinely used to discriminate early life stages are illustrated for *Pocillopora damicornis* and related *Sylophora pistillata* species in (Vandermeulen and Watabe, 1973; Gilis et al., 2014; Lecointe et al., 2016).

Although coral development is known to display a degree of plasticity, and metamorphosis was once proposed to be reversible, based on the reported ability of *Pocillopora damicornis* settled larvae to revert shape to swimming planula larvae in unfavorable reef environments (Richmond, 1984), transcriptome-based studies before/after metamorphosis have highlighted stage-specific transcriptional signatures in several coral species. Congruent gene expression patterns across metamorphosis were indeed revealed for aquarium-grown representatives of both major Robusta and Complexa clades of scleractinian corals (*Pocillopora*, *Stylophora*, *Acropora*, *Orbicella*, and *Porites* genera) (Grasso et al., 2008; Meyer et al., 2011; Hayward et al., 2011; Strader et al., 2018; Walker et al., 2019; Ishii et al., 2022; Reyes-Bermúdez and Rodríguez-Cabal, 2024; Zhang et al., 2024) indicating conserved mechanisms at transcript level. In a high temporal resolution study of *Acropora tenuis* (Ishii et al., 2022) a “Point of No-Return”, corresponding to metamorphosis commitment, was defined based on differentially expressed gene modules between planula and settlers (Ishii et al., 2022). Shifts in gene expression resulted in grouping together pre-metamorphosis swimming planula and thick disk-shaped, probing larvae [“stage 3” after Lecointe et al. (2016)], separately from metamorphosis-committed flat disk-shaped, settling larvae [“stage 4” after Lecointe et al. (2016)].

At protein level, the physiological processes involved during the metamorphosis transition are less clear. Transcripts may not be translated into proteins or exhibit a delay between life stages. As gene expression final products, proteins are therefore key biomarkers of physiological transitions during development. Yet the identity and respective roles of coral proteins enriched during metamorphosis remain underexplored, especially in the absence of exogenous microbial inducers.

The choice of the coral model is important to study this transition. For practical reasons, it is very important to choose a species which reproductive cycle is well characterized and synchronized, to collect enough planula larvae. In addition, the availability of genomic and transcriptomic data is a major criterion for the identification and annotation of protein sequences obtained by LC-MS/MS analysis (Wang et al., 2012). In this context, aquarium-grown representatives of *Pocillopora*, *Stylophora* or *Acropora* make good models for in-depth metamorphosis studies. The species chosen for this study was the scleractinian coral *Pocillopora acuta* Lamarck 1816, a widespread Indo-Pacific, pioneer reef-building species belonging to the species complex *Pocillopora damicornis* in the family Pocilloporidae. This species complex is known to have mixed spawning and brooding adaptive reproduction strategies, ensuring successful reproduction in various environments (Smith et al., 2019). Planula larvae may be produced both sexually and asexually via parthenogenesis from within the same colony (Combosch and Vollmer, 2013). In flow-through aquarium cultures supplied with natural seawater and suitable light, water flow and feeding, *Pocillopora acuta* Lamarck 1816 colonies brood and release Symbiodiniaceae *(Cladocopium*) - containing planulae several months per year (Océanopolis Brest, France (Kopp et al., 2016)), often synchronized with the full moon cycle (Lam et al., 2023). Such *ex-situ* model, where taxonomy of host *Pocillopora acuta* and symbiont *Cladocopium* was checked molecularly (Kopp et al., 2016), provides regular access to planulae competent to settle into primary polyps and budding recruits (Gilis et al., 2014; Massé et al., 2018).

Recent proteomic studies of contrasted *Pocillopora* coral life stages have mostly focused on the protein response of the coral holobiont (planula and adult) to experimental thermal and/or acidification stress, with equal attention to proteome changes in the animal host than in its endosymbiotic “microalgal” (photosynthetic dinoflagellates) Symbiodiniaceae fraction: they identified thermo-sensitive protein processes across planula and adult during environmental perturbation of the coral-algal symbiosis (McRae et al., 2021; Jiang et al., 2022; Sun et al., 2022). In settling larvae of *Stylophora* corals, a targeted proteome study, focused on biocalcification pathways, identified proteins from the calcified exoskeleton of corals (Akiva et al., 2018). Very recently, proteome changes were detected across *Pocillopora damicornis* larval stages (planula, metamorphosed, settled) exposed to a *Metabacillus indicus* bacterial strain inducer (Guo et al., 2025). Further untargeted studies, in the absence of bacterial challenge or thermal or acidification (exogenous) stress, are needed to investigate metamorphosis-related “baseline” (endogenous) proteome changes in coral larvae allowed to settle in a controlled laboratory environment. Indeed, a better understanding of the proteins supporting larval metamorphosis initiation, with identification of related biomarkers, will provide the foundation for a better knowledge of coral settlement processes, with potential future applications in population restoration strategies.

Our major goal was here to identify metamorphosis-related proteins in settling vs. swimming *Pocillopora acuta* Lamarck 1816 coral larvae, to characterize the coral phenotypic transitions at onset of metamorphosis, while assessing maternal carry-over effects. Additionally, to refine the spatial localization of key proteins involved in metamorphosis, we identified proteins enriched in aboral vs. oral planula tissue.

Planula larvae were collected from aquarium reared *Pocillopora acuta* Lamarck 1816 parent colonies during circalunar planulation events at Océanopolis, Brest, France. They spontaneously underwent metamorphosis *in vitro* in filtered seawater. Such controlled conditions (in the absence of exogenous microbial biofilm) allowed here to focus on the genetically encoded larval physiological changes, uncoupled from the complex responses to metamorphosis-inducing bacterial strains (Petersen et al., 2023; Guo et al., 2025) or natural microbial assemblages (bacteria and microeukaryotes associated to coralline crustose red algae, reviewed in (Jorissen et al., 2021; Turnlund et al., 2025)). Successive developmental stages were sorted out morphologically at onset of metamorphosis, discriminating swimming planula larvae (cylindrically shaped, undergoing reversible elongation/contraction along the oral/aboral axis) from early metamorphosis, stage 4 settling larvae [disk shaped, with irreversibly differentiated aboral calcifying basal plate and oral tentacle budding] as illustrated by (Lecointe et al., 2016) for Pocilloporidae corals. Early metamorphosis, stage 4 larvae were still in suspension (solitary or adhering laterally to each other), or they were lightly attached (detached under a gentle flux of seawater). When left uncollected, these settling larvae attached firmly to the dish surfaces and continued normal post-metamorphosis growth into adherent primary polyps and budding juveniles (Gilis et al., 2014; Massé et al., 2018).

Coral larval proteomes were analyzed using qualitative (presence/absence) and semi-quantitative (relative abundance) proteomics of two developmental stages, swimming planula (before metamorphosis) vs. settling larva (early metamorphosis, stage 4), and of bisected aboral vs. oral planula tissue.

Proteins were identified and differential abundance was assessed in paired treatment comparisons. Fold change was calculated using label-free quantification (LFQ) based on univariate statistical tests. Comparisons of data from 4 individual parent colonies allowed us to assess maternal carry-over effects on proteomes of planula and settling larva. In addition, comparisons of half-planula proteomes allowed us to address specialization at aboral pole, the area that probes and selects benthic microenvironments prior to larval settlement. Proteomic datasets were compared to transcriptomic datasets of oral/aboral halves of planula larvae of the same *Pocillopora acuta* Lamarck 1816 model (Ramon-Mateu et al., 2025). Implications of the detected proteome changes, in planula tissue and at metamorphosis onset, are discussed. A core set of larval proteins involved in coral metamorphosis regulation is proposed.

## Methods

### Biological material

The planula larvae released 5 days before full moon by 4 aquarium-propagated *Pocillopora acuta* Lamarck 1816 parent colonies (C3, C5, C6, C8) were collected in February 11–2022 at Oceanopolis, Brest, France. These colonies were obtained by long-term clonal sub-fragmentation from initial fragments imported from Indonesia in 2011, with potential cross-hybridization in the 10+ years period of aquarium propagation. Their genotypes were not characterized, but they were individually labeled and treated as distinct phenotypes. They shared one large ∼0.4 m3 aquarium holding tank filled with running natural seawater (4000 L.h^-1^ water flow, 10% daily renewal, pumped in Rade de Brest, pre-filtered mechanically and biologically) heated to ∼25 °C, pH ∼ 8.2. Colonies were fed with *Artemia* nauplii three times per week, under 12 h:12 h day/night photoperiod provided by 2 HQI lamps (Spot pro Let 200W Corallien DMX) and ambient natural skylight providing 200–300 μmol.m^-2^.s^-1^. They were placed at distinct positions relative to light and in- and out-flow, likely to influence their individual nutritional status and maternal investment in offspring (brooded planula). Reproductive cycling was asynchronous, with between-colony variability in planula abundance and time to peak planulation (11 Feb 2022, for C3, C5 and C8, delayed for 1 day for C6). Larvae were collected and transported separately by individual parent colony origin, in filtered aquarium seawater (Minisart® Sartorius Syringe Filter PES 0.22 µm) to the MCAM laboratory in Paris, France, where planulae were immediately sampled (in pools of 10 larvae per replicate per parent colony, on 11 Feb 2022) and stored at −20 °C. The rest of the planulae were allowed to spontaneously develop *in vitro*, at densities of 50–75 larvae/250 mL filtered seawater (50% renewal every 2 days) for 5 days (C3, C5) to 6 days (C6, C8) at 25 °C, in orbital shaking incubator (Infors, Switzerland; 40 rpm), with a 12 h:12 h day/night photoperiod and ∼50–100 μmol.m^-2^.s^-1^ irradiance (4 Osram TBL15W/840Active/4000 K CoolWhite light). Larvae were sorted morphologically under a binocular dissecting microscope by developmental stage, into either planula larva (elongated/contracted cylinder) or “stage 4” settling larva (flattened disk-shaped larva, with visible calcified elements and tentacle buds) (illustrated in Supplementary Figure S1). Commitment to metamorphosis in settlers (stage 4) was confirmed by lack of relaxation to planula shape when exposed to 7.5% MgCl_2_. The settlers (stage 4) were sampled in pools of 10 larvae per replicate for every colony origin in 2 ml Eppendorf tubes and stored at −20 °C. Each planula/settler group was replicated 3–4 times per each of 4 parent colonies, resulting in 13 extracted pools of 10 larvae per development stage and a total of 26 samples (Supplementary Figure S1; Supplementary Table S1).

Planula larvae of the same *Pocillopora acuta* Lamarck 1816 parent colonies were also collected 1 year later in February 2023 and were dissected 5 days post-planulation into aboral and oral halves using a scalpel under a binocular. Aboral and oral halves (poles) were sampled in pools of 3-4 half-larvae per replicate per parent colony, in 2 ml Eppendorf tubes and stored at −20 °C. Each aboral/oral group was replicated 3 times across parent colonies resulting in a total of 6 samples (Supplementary Figure S1; Supplementary Table S1).

### Experimental design

#### Sample preparation

Proteins were extracted from the larval samples with a Trifluoracetic acid (TFA) extraction protocol adapted from the protocol of Doellinger et al. (2020). Briefly, 50 µl of TFA (100%) was added to each Eppendorf tube containing 10 larvae, incubated for 5 min at room temperature and sonicated (Advantage-Lab™ AL04-04–230 at frequency of 50/60 Hz and 280 W power), for 3 to 10 cycles of 20 s on ice until complete tissue disruption. The extract was neutralized with 350 µl of 2 M Tris base (pH∼8). The proteins were reduced and alkylated by adding 55 µl of 100 mM tris(2-carboxyethyl)phosphine (TCEP) followed by 55 µl of 400 mM chloroacetamide (CAA) at 95 °C for 5 min. To facilitate further treatment, samples were divided into 2 equal volumes (255 µl) and diluted with 1,125 µl of MilliQ water. Proteins were digested by adding 1 µg of Trypsin Gold (from Promega, United States) overnight at 37 °C. Digestion was stopped by adding 22.5 µl of pure TFA. Digested proteins were then desalted using Pierce™ C18 Spin Columns from Thermo Scientific™, then evaporated to nearly 5 µl in a SpeedVac and finally resuspended in 20 µl of 2% acetonitrile (ACN) 0.1% TFA. The samples were then stored at −20 °C until liquid chromatography-tandem mass spectrometry (LC-MS/MS) analysis. Protein concentrations were measured using a bicinchoninic acid (BCA) assay kit (Pierce BCA Protein Assay Kits, ThermoFisher).

The same protocol was used for the bisected larval samples. Due to lower biomass of bisected planulae (3-4 half larvae pools) than whole planulae/stage 4 (10 larvae pools), protein concentrations were quantified only in whole larvae. Only samples yielding similar numbers of detected proteins were selected. The detailed sample list and corresponding LC-MS features are provided in Supplementary Table S1.

### LC-MS/MS data acquisition

A volume of 2 µL of the peptide digests was injected and concentrated on a C18 cartridge (Dionex Acclaim PepMap100, 5 μm, 300 µm i.d. × 5 mm) and eluted on a capillary reverse-phase column (nanoEase M/Z Peptide CSH C18 Column, 130Å, 1.7 µm, 75 μm × 250 mm) at 220 nL/min, with a gradient from 2% to 40% of buffer B in 45 min (A: 0.1% aqueous formic acid/ACN 98:2 (v/v); B: 0.1% aqueous formic acid/ACN 10:90 (v/v)), coupled to a quadrupole-Orbitrap mass spectrometer (Q Exactive HF, ThermoFisher Scientific) with electrospray ionization in positive mode using a Top 20 data-dependent acquisition MS experiment: 1 survey MS scan (400–2,000 m/z; resolution 70,000) followed by 20 M/MS scans on the 20 most intense precursors (dynamic exclusion of 30 s, resolution 17,500). Nano-LC-MS/MS data were generated in the Proteomics platform at ESPCI, Paris, France.

### Data analyses

All the raw liquid chromatography tandem mass spectrometry (LC-MS/MS) data were first searched using PEAKS Xpro 10.6 (Bioinformatics Solutions Inc, Canada), and a reference database of 787913 Cnidaria protein sequences downloaded from NCBI (download date: 07/04/2024). This reference database focuses on metazoan host (Cnidaria) proteins, excluding the proteins of Symbiodiniaceae dinoflagellate endosymbionts. Alternately a Symbiodiniaceae reference protein database (895379 sequences) was downloaded from NCBI (download date:14/05/2025) to search the LC-MS/MS dataset for dinoflagellate proteins across both larval development stages of the *Pocillopora acuta* coral holobiont, as this aquarium-reared brooding coral model species is known to associate with vertically transmitted, mostly *Cladocopium* sp. dinoflagellates (Kopp et al., 2016).

Following search parameters were used for protein identification: parent mass error tolerance: 10 ppm, fragment mass error tolerance: 0.05 Da, enzyme: trypsin, max missed cleavages: 3, digest mode: semi specific. Carbamidomethylation of cysteine was set as the fixed modification; deamidation (NQ and oxidation of Methionine were chosen as variable modifications); False Discovery Rate of (FDR) for protein identification was <1%, LC-MS/MS data were also searched using FragPipe V22.0 with MSFragger v 4.1 and IonQuant v1.10.27 (Hsiao et al., 2024) with the following parameters: data_type: Data Dependant Acquisition (DDA), precursor_true_tolerance: 20 ppm, fragment_mass_tolerance: 20 ppm, enzyme: trypsin, digest mode: semi specific; ion quantification with Match Between Runs (MBR); intensity normalized across runs; FDR <1%.

The results were filtered to eliminate common proteomic contaminants (using a contaminant database implemented in PEAKS or FragPipe workflows), then further filtered to retain only proteins with 2 unique matching peptides (PEAKS) or only proteins with unique plus razor peptides (FragPipe). The mass spectrometry proteomics data have been deposited to the ProteomeXchange Consortium via the PRIDE partner repository (Perez-Riverol et al., 2025) with the dataset identifier PXD071713.

The variability of larval protein profiles (each sample is a pool of 10 larvae) was visualized across 2 development stages (before/during metamorphosis) and 4 parent colony origins using a PHATE (Potential of Heat-diffusion for Affinity-based Trajectory Embedding) method for dimension reduction and estimation, with *Rdimtools* R package (You and Shung, 2022). To show the variability between oral and aboral protein profiles, a Principal Component Analysis considering the top 500 features was performed.

Venn diagrams were built with Venny 2.1 tool (Oliveros, 2020) to visualize shared and unique proteins across the proteome datasets of planula versus stage 4 larval stages, and across the 4 parent colony origins within each stage, and also between the planula oral and aboral pole tissue proteomes.

To highlight differentially abundant proteins (referred to as DEPs), significantly enriched at the onset of *Pocillopora acuta* coral metamorphosis, in settling compared to planula larvae, and between aboral and oral poles, Label Free Quantification (LFQ) was performed using FragPipe-Analyst (Hsiao et al., 2024). Differentially abundant proteins (DEPs) were detected based on their Fold Change (log2 FC ratio St4/planula > |2|, aboral/oral > |2|), and adjusted p-values <0.05 with Benjamini Hochberg correction.

For annotation of unknown/uncharacterized DEPs, their sequences were blasted using the BLASTP algorithm (protein-protein BLAST (Altschul et al., 1990), in NCBI with an E-value <10^−5^ to find homologous sequences from which predicted functional annotation could be assigned. Additional search for domains was conducted with InterProScan tool implemented in OmicsBox 3.3 (Jetzinger, 2024) for classification into protein families.

Gene Ontology analyses were performed using OmicsBox 3.3 (Jetzinger, 2024) to classify DEPs based on their molecular function (MF) and functional domains. Annotated DEPs were also clustered into broad functional categories, predicted from combined GO and InterProScan domain results, and inferred from the published functions of best matching hit (BLASTP) proteins. This approach was used to highlight blocks of proteins involved in specific metabolic pathways, upregulated or downregulated at onset of metamorphosis.

## Results

### Overview of identified coral proteins across larval metamorphosis stages

In this work, we focus on the coral host proteins and their variation at the onset of larval metamorphosis (swimming planula to settling larva transition). In addition, results for the endosymbiotic Symbiodiniaceae proteins are provided at the end of each Results subsection.

**FIGURE 1:**
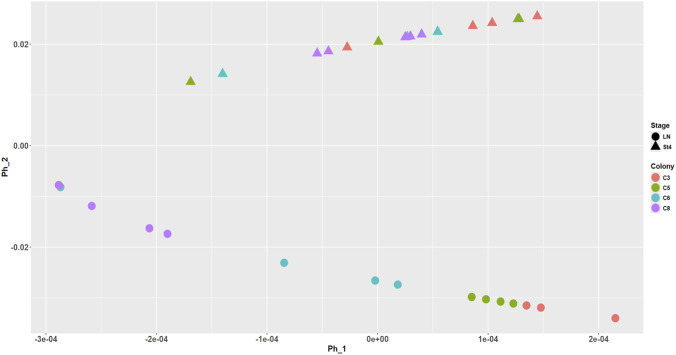
PHATE model representing the proteomic profiles distribution between the 2 developmental stages, settling larva (St4) and planula (LN), and across the 4 different parent colony origins. Proteome profiles were compared with discriminant analysis of protein intensities taking into account merging of dummy variables, which correspond to development stage and colony factors. The principal component Phate 1 represents a scaling factor that highlights differences in proteome distribution among the four colonies, both at the planula and settling larva stage. Component 2 reveals a difference in the average proteome distribution between planula and settler.

PHATE analysis (see Methods) revealed a clear difference in the proteomic signatures between settling (st4) and planula larvae, and also the influence of the different parent colony origins. Indeed, the Phate 1 component (Figure 1) represents a scaling factor that highlights differences in proteome distribution among the various colonies, both at the planula and settling larva stage. Phate 2 component reveals a clear difference in the average proteome distribution between planula and settler.

The cumulated proteomes of all planula (n = 13) and stage 4 (n = 13) samples, analyzed with FragPipe (see Methods), contained a total of 45978 peptides corresponding to 5,570 coral proteins, which were distributed almost equally in planula (4,408 proteins) as in settler (4,198 proteins). Among these coral proteins, a majority (54.5%, 3,036 proteins) were shared between the two stages with only 20.9% and 24.6% specific to each stage, respectively. Despite high inter-parent colony variability, about 30% of planula and 25% of settler’s proteins were shared (Supplementary Figure S2). Each protein extract contained an average of 2578 ± 338 [1826–3,129] coral proteins (mean ± SD, range, n = 26) (Supplementary Table S1).

In addition, about 700–800 Symbiodiniaceae proteins were detected in larvae across both stages, corresponding to the proteome fraction of the endosymbiotic dinoflagellates, with an average of 644 ± 118 [419–794] Symbiodiniaceae proteins per extract (mean ± SD, range, n = 26) (Supplementary Table S1). Overall, the holobiont proteome was composed of ∼20% algal proteins and ∼80% coral proteins.

At each development stage, high variability in larval proteomes was illustrated in the Venn diagrams (Supplementary Figures S2B,S2C), confirming the separation of protein profiles by parent colony origin on Component 1 of the Phate plot (Figure 1) and highlighting the importance of the parent colony factor in the heterogeneity of larval protein phenotypes. To avoid masking the detection of changes in protein expression across stages by the large variability due to parent colony origin, subsequent larval proteomes were thus analyzed by pairwise comparison between development stages for each individual parent colony origin (n = 3-4 replicates × 2 stages). The results obtained for the 4 colony origins were then combined for visualization (Supplementary Figures S2,S3; Supplementary Table S2).

### Differentially abundant proteins (DEPs) enriched in settler (St4) or in planula

**FIGURE 2:**
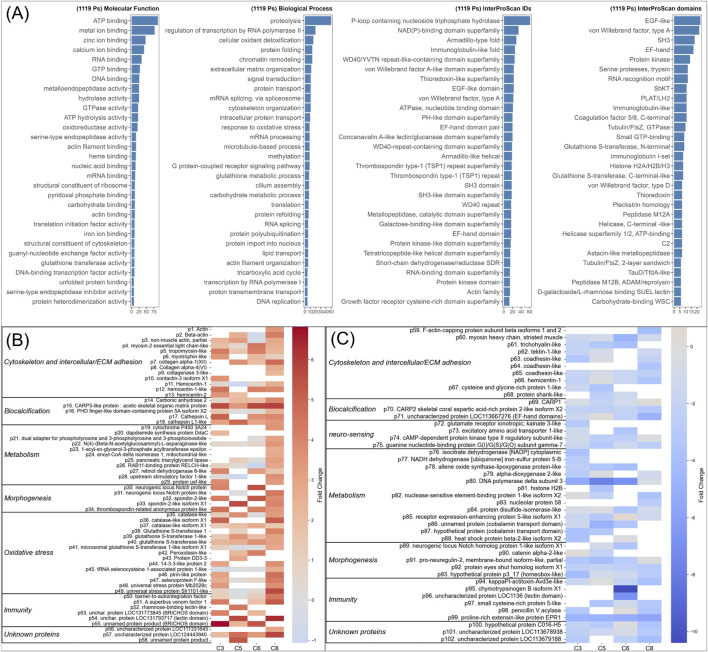
Differential occurrence and enrichment of coral proteins at onset of metamorphosis **(A)** Functional annotations associated with the 1,119 quantified proteins showing differential presence/absence between planula and settler (combined FragPipe results of 4 pair-wise comparisons of larval proteomes per colony origin): top 30 Gene Ontology (GO) terms for Molecular Function (MF) and Biological Process (BP), and top 30 InterProScan Identifications (domain, family, and repeats) with a focus on top InterProScan domains. Details of protein sequences and annotations, filtered by development stage (planula: 827, St4: 329), are provided in Supplementary Table S3 **(B,C)** Heatmaps of the 102 significant differentially enriched proteins (DEPs), with color-coded relative abundance levels (log_2_ fold-change ratios between St4 and planula). Detailed annotations and abundance levels are provided in Supplementary Table S2. Proteins are grouped into broad functional categories by predicted, literature-based biological function, including cytoskeleton and extracellular matrix (ECM) adhesion, biocalcification, neuro-sensing, metabolism, morphogenesis, oxidative stress protection, and immunity **(B)** Proteins enriched in settling larva (St4) **(C)** Proteins enriched in planula.

Qualitative analysis on the presence/absence (occurrence) of coral proteins (presence in at least one replicate of 1 stage/absence in all replicates of the other stage) highlighted a total of 1,119 stage-specific coral proteins, distributed mainly in planula (792) and to a lesser extent in settler (290) (completed by 37 proteins present in replicates of either planula or settler, depending on parent colony). Label-free quantification across the two stages revealed a subset of 102 coral DEPs (quantified in all replicates of one stage with absolute fold change of log2 ratio >2 and p-value <0.05), which are graphically illustrated in Volcano plots per every parent colony (Supplementary Figure S3) and visualized in a heatmap of color-coded FC values. Among the detected coral DEPs, 58 were enriched (Figure 2B) and 44 were depleted (Figure 2C) in settlers compared to planulae. The numbers of DEPs varied across larval parent colony origins, with most identified in C8 (58; Supplementary Figure S3D), least in C5 and C6 (21 and 17, respectively; Supplementary Figures S3B,S3C) and an intermediate number in C3 (32; Supplementary Figure S3A). The detailed annotations of the 102 DEP sequences (UniProt E-values <10^−5^, InterProScan IDs) is provided in Supplementary Table S2. Only 6 DEP sequences were not annotated (3 unknown DEPs per stage).

Regarding Symbiodiniaceae proteins, LFQ analysis revealed only 6 DEPs (|FC| >2, p-value <0.05), among which 2 were enriched and 4 depleted in settlers (St4) relative to planulae (4 DEPs/803 Symbiodiniaceae proteins in C8; 2/1,023 in C5; none detected in C3 or C6). Identities of the 6 Symbiodiniaceae DEPs are provided in Supplementary Table S2. Overall, the Symbiodiniaceae response to metamorphosis is weak as indicated by the lesser number of proteins modulated in algae symbionts compared to coral hosts.

The number of larval DEPs showed high variability depending on the parent colony. Venn plots of 102 coral DEPs indicates that the majority (79/102) of strongly significant DEPs (|FC| > 2, p-value <0.05) were detected in larvae from a single colony; 18 DEPs were shared between larvae of 2 parent colonies; 4 DEPs were shared between larvae of 3 parent colonies [planula-enriched DNA polymerase delta subunit 3 (PFX18529.1) and allene oxide synthase-lipoxygenase protein-like (XP_027058640.1); settler’s enriched CARP3-like protein: acidic skeletal organic matrix protein (XP_058960654.2) and spondin-2-like (XP_058954575.1) ]; only 1 DEP [planula-enriched proline-rich extensin-like protein EPR1 (XP_022780794.1)] was shared between the 4 parent colonies. However, most of the proteins identified as DEP in larvae of at least one colony (FC values highlighted in bold, Supplementary Table S2), were also detected in larvae of other colonies, although at intensities below significance levels (Supplementary Table S2). At protein group level, similar relative abundance patterns were shared across parent colonies, with intensities that varied between colonies. To summarize, the diversity and amplitude of protein response to metamorphosis in larvae depended on parental origin. The offspring of parent colony C8 displayed a stronger metamorphosis response than the offspring of the 3 other parent colonies, with most differentially enriched coral proteins.

### Protein domains, molecular functions and biological process enriched in settling vs. planula larvae

Gene Ontology (GO) and domain/repeats/family of coral proteins enriched during metamorphosis is illustrated in Figure 2A, indicating the top 30 GO terms for molecular function (MF) and biological process (BP) and top30 InterProScan identifications (repeats, family IDs) and domains, assigned to the 1,119 quantified proteins that are present/absent in planula or settlers (detailed annotations per development stage provided in Supplementary Table S3). Most enriched GO biological processes (Figure 2A) are related to proteolysis (50 seq) and transcription (regulation by RNA polymerase II, 20 seq and RNA polymerase I, 5 seq; chromatin remodeling, 14 seq; mRNA splicing, 11 seq; mRNA processing, 9 seq). Metamorphosis also involved antioxidant capacity (with GO terms assigned to cellular oxidant detoxification, 16 seq; response to oxidative stress, 9 seq; gluthathione metabolic process, 8 seq). Active protein translation, maturation and transport were also detected (GO terms assigned to translation, 7 seq; protein folding, 14 seq; refolding, 7 seq; transport, 11 seq). Additional enriched biological process included reorganization of extracellular matrix (12 seq) and cytoskeleton (11 seq), and signal transduction (11 seq), including G protein-coupled receptor signaling (9 seq). Metabolism of stored carbon energy was also active (carbohydrate metabolism, 8 seq; tricarboxylic acid cycle, 5 seq). Corresponding enriched molecular functions (MF) (Figure 2A) were dominated by GO terms related to metal ion binding (metal, 78 seq; zinc, 47 seq; calcium, 42 seq; iron, 10 seq), nucleic acid binding (RNA, 34 seq; DNA, 26 seq), and GPCR signaling activity (GTP binding, 28 seq; GTPase, 22 seq; and guanyl-nucleotide exchange factor activity, 9 seq). Additionally, terms related to hydrolase (25 seq.) and endopeptidase activities (metallo-type, 25 seq; serine-type,15 seq) activities were enriched, supporting ATP energy production (ATP hydrolysis, 25 seq) and proteolysis (see BP above), to fuel cytoskeleton remodeling (actin filament binding, 15 seq; structural constituent of cytoskeleton, 9 seq), redox cycling (oxidoreductase activity, 20 seq) and oxidative stress protection (glutathione transferase activity, 9 seq; peroxidase activity, 7 seq).

Congruent with these findings, top represented domains, repeats and families during metamorphosis (Figure 2A) included P-loop containing nucleoside triphosphate hydrolase (61 seq) involved in ATPase and GTPase activities, and protein-peptide (Armadillo-type fold, 29 seq; Armadillo-like helical 19 seq; SH3 18 seq and SH3-like 18 seq) and protein-protein interaction domain (WD40 repeat 25 seq, von Willebrand factor A -like domain 23 seq) superfamilies involved in multiprotein complexes for signal transduction pathways. Additional enriched protein families included oxidoreductases containing NAD(P)-binding domain superfamily (34 seq), as well as immunoglobulins (immunoglobulin-like fold, 26 seq; Immunoglobulin-like domain superfamily, 12 seq) and lectins (concanavalin A-like lectin/glucanase domain, 20 seq.; galactose-binding-like domain, 17 seq.). The thrombospondin type-1 (TSP1) repeat superfamily (18 seq.), and several peptidase (MB12A, M12B ADAM) and metallopeptidase domains were also detected among the 1,119 differentially occurrent proteins. The TauD/TfdA domain (6 seq.) involved in taurine catabolism (GO term oxidoreductase activity), was equally detected in planula (3 seq.) and settler (3 seq.), including in the settler enriched dapdiamide synthesis DdaC-like protein (XP_058943065.1).

In the subset of 102 DEPs identified in settler vs. planula, corresponding to proteins which abundance strongly varied during metamorphosis, the enriched GO terms and Interproscan IDs are shown in Supplementary Figure S4. The six most represented biological process (BP) categories were anatomical structure development (6 seq.), sulfur metabolism and cell adhesion (5 seq. each), followed by transcription, lipid metabolism and cell differentiation (4 seq. each). Six molecular function (MF) categories were represented by more than 3 proteins, highlighting oxidoreductase (11 seq.) and hydrolase (8 seq.) activities, followed by transferase activity, DNA binding, catalytic activity on a protein, and antioxidant activity (4 seq. each). Top represented InterProScan domains, repeats and families were related to protein interactions with ligands (von Willebrand factor A domain superfamily, 6 seq.), TSP1 repeat superfamily (6 seq.), growth factor related EGF-like domain (6 seq.) and calcium-binding sites (EF-hand domains, 6 seq.) associated to morphogenesis signaling. Immunity-related proteins were also strongly represented among the 102 DEPs, such as immunoglobulins (Immunoglobulin-like fold, 4 seq.; Igl-set, 3 seq.) and lectins (concanavalinA-like domain, 4 seq.; galactose-binding-like domain, 3 seq.). Moreover, antioxidant defense proteins were upregulated, as shown by enriched glutathione S-transferase (C-terminal and N-terminal) like domains (6 seq. each), and multiple catalase domains (haem-containing, 3 seq.; core, 3 seq.; immune-responsive, 3 seq.) as well as the thioredoxin-like superfamily (5 seq.).

### Stage-specific enriched putative functions

Protein groups, clustered based on sequence variants and similar identities in related coral taxa (e.g., actins from *Stylophora* and *Pocillopora*), were assigned to putative functional categories, inferred from literature on the function of the detected proteins, domains, and repeats (e.g., immunity-related clustering of proteins containing Immunoglobulin and lectin domains). Globally, 8 putative functional categories were defined to classify the 102 differentially enriched proteins across stages. These categories corresponded to cytoskeleton and intercellular/ECM adhesion, neuro-sensing, biocalcification, metabolism, morphogenesis, immunity, and unknown (not annotated). An additional category, oxidative stress protection, was represented only in DEPs of settlers and not in those of the planulae.

The 58 proteins significantly enriched in settlers were clustered into 7 predicted functional categories (Figure 2B) classified from top to least represented as follows: 1. Oxidative stress defense proteins (15 proteins, including multiple ROS scavenging proteins such as 4 glutathione transferases, 3 catalases, a SOD domain-containing protein DD3-3, a peroxidasin, and 2 selenoproteins); 2. Cytoskeleton and intercellular/ECM adhesion (13 proteins, including myosin-2 essential light chain, tropomyosin-like and myotrophin-like proteins, a contactin intercellular adhesion and neural recognition molecule, plus 3 hemicentins); 3. Metabolism (11 proteins, including mitochondrial enzymes, lipid catabolism and transport proteins (including endosomal recycling RAB11-binding protein XP_027047557.1), a cytochrome P450 3A isoform, and the TauD domain containing oxidoreductase dapdiamide synthesis protein DdaC-like (XP_058943065.1); 4. Immunity (6 proteins, including 2 lectin-domain and 2 BRICHOS domain containing proteins, the latter with predicted antimicrobial peptide activities (Supplementary Figure S5 (Veltri et al., 2018; Safronova et al., 2024)); a neurotoxin and a barrier-to-autointegration factor); 5. Biomineralization (5 biocalcification toolkit proteins, including the coral acid-rich skeletal matrix protein CARP3, a carbonic anhydrase 2, and 2 cathepsin L proteases); 6. Morphogenesis signaling (5 proteins, including 2 neurogenic Notch and 3 spondin-like proteins); 7. Unknown proteins (3 proteins).

The 44 proteins significantly enriched in planulae (depleted in settlers) were clustered into 7 predicted functional categories (Figure 2C) corresponding to: 1. Metabolism (13 proteins, attributed to energy metabolism, arachidonic acid metabolism, cell cycling, vitamin transport and intracellular trafficking); 2. Cytoskeleton and intercellular/adhesion (10 proteins, including 3 co-adhesins, 2 cilium structural proteins and 1 hemicentin-1); 3. Immunity (6 proteins, including a proline-rich extensin-like neurotoxin, a Kunitz-type venom, a small cysteine-rich protein, a lectin-domain protein, chymotrypsinogen (protease precursor), and a penicillin acylase enzyme interfering with quorum-sensing of environmental microbes); 4. Morphogenesis (5 proteins, including a Notch-1 protein, a pro-neuregulin-2, a homeobox-like hypothetical protein, and a catenin alpha-2-like protein); 5. Neuro-sensing (4 proteins, including a glutamate ionotropic receptor, an excitatory amino acid transporter and 2 GPCR-signal transduction proteins); 6. Biomineralization (3 biocalcification toolkit proteins including a calcium-binding EF-hand domain protein and 2 different CARP1 and CARP2 than in settlers i.e.); 7. Unknown proteins (3 proteins).

Proteome differences between development stages were both qualitative and quantitative. Each predicted functional category contained distinct identities and numbers of present/absent or differentially enriched proteins depending on stage.

For example, proteins of the GPCR signaling pathway were enriched in planula (12 sequences, including adrenergic beta-1 (CAH3117642.1) and tachykinin (CAH3043180.1) neuropeptide receptors, adhesion G-protein coupled receptor (XP_058951762.1)) compared to settlers (only 2 isoforms of Guanine nucleotide-binding protein G(I)/G(S)/G(O) subunits gamma KAJ7383655.1 and XP_027054344.1). Likewise, proteins with TSP1 repeats were enriched in planula (13 sequences including 4 ADAMTS metalloproteinases, 5 hemicentin-1 like proteins, 1 co-adhesin, and 1 notch-1 protein) compared to settlers (5 sequences, including 1 ADAMTS protein, 2 hemicentin-1 like proteins, 1 co-adhesin). Moreover, the identities of intercellular adhesion-related hemicentin variants, biocalcification-related CARP proteins, and immunity-related toxins/venoms and lectins, were different before (in planula) and during metamorphosis (in settler), indicative of different biological functions. Importantly, proteins classified to the functional category oxidative stress protection were dominant among DEPs upregulated in settlers (stage 4), corresponding to the most represented function at metamorphosis onset.

### Overview of identified proteins in aboral and oral planula halves

**FIGURE 3:**
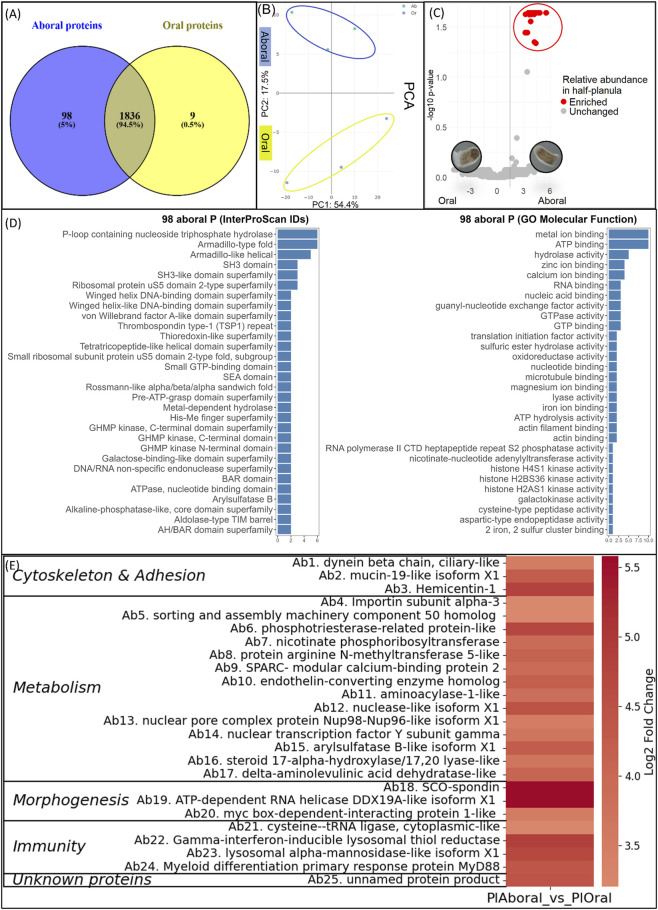
Aboral enriched coral planula proteins **(A)** Venn diagram showing the number of shared and unique identified proteins in bisected planula larvae: 1836 proteins are common to both oral and aboral halves; 9 proteins are uniquely present in the oral pole tissue; and 98 proteins are uniquely present (73) or differentially enriched (25) in the aboral pole tissue **(B)** Principal Component Analysis of planula aboral (blue) and oral (yellow) proteome profiles **(C)** Volcano plot illustrating the differential protein abundance between aboral and oral halves. The x-axis represents the log2 fold change (Aboral vs. Oral), and the y-axis shows the − log10 adjusted p-value. Black points indicate differentially abundant proteins between the two poles, revealing 25 aboral enriched proteins and none orally **(D)** Gene Ontology [Molecular Function] terms and InterProScan domain, repeat, and family distribution associated with the 98 aboral proteins **(E)** Heatmap of the 25 differentially enriched aboral proteins. Enrichment values are the log2 fold change ratio between aboral and oral halves.

The proteome dataset of half-planula extracts contained a total of 8,850 peptides corresponding to 1943 proteins (Supplementary Table S1). Spatial differences between oral and aboral planula proteomes, visualized in Venn diagram (Figure 3A), can be summarized as following. Half-planula samples contained a total of 1836 (94.5%) shared proteins (common to both oral and aboral planula halves) with only 0.5% (9 oral) to 5% (73 aboral) proteins specific to each pole. Each half-planula extract contained an average of 1,517 ± 311 [975–1829] coral proteins (mean ± SD, range, n = 6) (Supplementary Table S1). Proteomes of oral and aboral half-planula clustered separately as shown via Principal Component Analysis (Figure 3B).

Label-free analysis between aboral and oral planula halves revealed 25 differentially enriched proteins in aboral tissue and none in oral tissue (DEPs, Figure 3C).

For the 98 proteins enriched in or unique to the aboral pole tissue (25 DEPs and 73 proteins present only aborally) gene ontology annotation (Figure 3D, detailed in Supplementary Table S4) highlight 2 main molecular functions that were assigned to more than 10 proteins i.e., metal ion binding and ATP binding. In addition, hydrolase activity was assigned to more than 5 proteins (including energy-producing ATP hydrolysis activity). Aboral planula proteins were significantly enriched in molecular function terms such as guanyl-nucleotide exchange factor/GTPase/GTP-binding activities, supportive of G Protein coupled receptor signaling. An aboral rhodopsin-like GPCR (PFX23700.1) was detected, via its 7TM 1 domain (IPR017452). Other aboral-enriched molecular functions were related to sulfur-binding and sulfur metabolism, peptidase, and oxidoreductase activities. The top 3 InterProScan terms (domain, repeat, superfamily; Figure 3D) assigned to the 98 proteins enriched in or unique to the aboral pole tissue, were P-loop containing nucleoside triphosphate hydrolase family (supportive of ATP consumption and GPCR signaling), and peptide-binding protein scaffolds involved in beta-catenin canonical intracellular signal transduction (Armadillo-type fold and Armadillo-like helical families; SH3 domains). Thrombospondin type 1 (TSP1) repeats were abundant in aboral proteins (Figure 3D) as well as domains and families of small metabolite kinases involved in amino-acid and galactose metabolism (GHMP kinase superfamily, galactose-binding domain and galactokinase activity). The SEA domain was detected in aboral protein sequences, that may have carbohydrate regulated proteolytic activity. The TauD domain (2 seq.) involved in taurine catabolism (oxidoreductase) activity was also detected amongst planula aboral proteins.

For the 9 proteins unique to the oral pole tissue (present only in oral and not aboral halves), assigned gene ontology molecular functions and Interproscan terms highlight peptidase, peroxidase and glutathione S-transferase domains and protein heterodimerization function (Supplementary Figure S6B).

The relative abundance of the 25 aboral DEPs was visualized by a heatmap (Figure 3E; annotation detailed in Supplementary Table S2; GO[Molecular Function] terms and InterProScan domain, repeat, and family distribution in Supplementary Figure S6A) and they were classified into 5 broad putative functional categories inferred from literature on the detected proteins and domains: 1. Metabolism (14 proteins, including amino-acid and fatty acid metabolism proteins, peptidases, endonucleases, intracellular trafficking proteins, steroidogenic CYP17A1-like alpha-hydroxylase/lyase, arylsulfatase-B sulfotransferase, and porphobilinogen biosynthetic delta-aminolevulinic acid dehydratase); 2. Immunity (4 proteins, including 2 lysosomal enzymes); 3. Cytoskeleton and adhesion (3 proteins, including, dynein ciliary motor protein, mucin19-like isoform and TSP-1 repeat -rich hemicentin-1); 4. Morphogenesis (3 proteins, including SCO-spondin, ATP-dependent RNA helicase (vasa-like) and myc-box interacting protein); 5. Unknown proteins (1 protein).

Overall, at tissue level, the planula aboral proteome was more specialized than the oral proteome. In addition, some aboral enriched planula proteins and domains (e.g., hemicentin-1 proteins and glutathione S-transferase domains) were also enriched in the proteomes of settlers (St4) relative to planulae, indicative of upregulation during metamorphosis initiation.

## Discussion

Here we present the proteomic signatures of two early life stages of the coral *Pocillopora acuta* Lamarck 1816, i.e., planula and metamorphosis-committed (stage 4) larva, and reveal significant changes in coral larval proteomes, highlighting proteins differentially enriched during metamorphosis. Additional proteome analysis of bisected planulae, i.e., oral vs. aboral halves, provides complementary spatial information at tissue level on aboral protein specialization before metamorphosis. Larval protein expression dynamics are integrated across these temporal and spatial scales, to provide an overview of physiological pathways activated at the very onset of coral metamorphosis.

### Strengths and limits of the experimental design

The aquarium experiment was designed to focus on larval protein response at settlement initiation, considering several variability factors. First, the use of larvae from four distinct *Pocillopora acuta* parent colonies allowed us to account for a parental effect. Colonies displayed distinct phenotypes due to potential parental genotype heterogeneity, and to nutritional status adjustments to microscale variability in light irradiance and water flow within the shared holding tank (see Methods). Their reproductive cycling was asynchronous, with sexual and/or asexual production of planulae (larval genotype heterogeneity), and likely distinct maternal investment in offspring (planula) performance. Between colonies, freshly released planula larvae were collected on the same date (planulation day) but at distinct times to planulation peak, and the timing of larval release within a single reproductive event is known in *Pocillopora damicornis* to influence larval competency to settle (Putnam et al., 2020). Additional variability in larval proteomes can also be attributed to asynchronous larval development, which prevents accurate time-based sorting, and leads to potential small development differences across morphologically sorted batches of 10 larvae (biological replicates). Indeed, the settling larva, “St4” stage (sampled 5–6 days post-planulation), grouped metamorphosis-committed larvae (validated through negative relaxation response to MgCl_2_ exposure) that may include early, middle, and late intermediate stages. Altogether, the proteome variability across pools of each development stage, before/after metamorphosis onset, is structured by multiple factors, and replication (3-4 biological replicates per parent colony origin and stage) was included to promote robustness in the identification of differentially enriched proteins.

Our experimental design nevertheless has some limitations. Intermediate developmental stages between planula and stage 4, and the later “primary polyp” post-settlement stage, were not included, and this restricts the analysis of temporal protein abundance dynamics during metamorphosis. However, pooling planula and stage 3 (“before metamorphosis”) separately from stage 4 and primary polyp (“after commitment to metamorphosis”) is justified by evidence of a transcriptional “Point of No-Return” separation between these groups in *Acropora tenuis* larvae (Ishii et al., 2022). Also, the oral/aboral tissue dissection experiment was limited to the planula stage, and did not include the parent colony origin factor. Miniaturization could be improved in future studies: here, proteomics was performed on pools of 10 *Pocillopora acuta* larvae (or 3-4 half larvae for bisected aboral/oral planula) instead of previously published pools of 20 larvae (McRae et al., 2021) or 100 larvae (Sun et al., 2022). Further miniaturization is recommended, because pooling larval samples limits the ability to detect differences between individuals.

### Proteomic evidence of planula aboral tissue specialization

The spatially resolved aboral tissue phenotype obtained via LFQ-proteomics (dataset of 98 unique/differentially enriched planula aboral proteins) was consistent with recent aboral RNAseq results obtained on *Pocillopora acuta* planulae (126 *Pocillopora* aboral DEGs (Ramon-Mateu et al., 2025). Compared gene and protein expression patterns (DEGs/DEPs) showed shared aboral enrichment in functional domains related to the extracellular matrix and cell adhesion (TSP1, von Willebrand, F5/8), as well as proteolysis (peptidases) and signaling/adaptor proteins (SH3). Protein domains involved in G protein coupled receptor (GPCR) signaling were also enriched both in planula aboral transcriptomes and proteomes. Overall, there was an overlap in domains associated with adhesion/extracellular matrix, proteolysis, and intracellular signaling. A mucin-19-like isoform (XP_027046324.1) was identified at both transcript and protein levels in planula aboral tissue. Other proteins, such as metalloproteinases (e.g. ADAMTS domains of metallopeptidases), were already overexpressed in planula aboral transcriptome, but enriched in larval proteome only at settler stage, illustrating a temporal shift between gene transcription and protein translation.

### Developmental shifts in ciliary, adhesion and ECM proteins highlight candidate aboral proteins involved in settlement initiation

Several planula enriched proteins were strongly depleted in settler (metamorphosis-committed larva), including ciliary (trichohyalin and tektin-1-like) proteins, co-adhesins (3 proteins), a neurosensory glutamate receptor ionotropic (kainite-3-like), an excitatory amino acid transporter and 2 G-Protein Coupled Receptor (GPCR) signaling pathway proteins. This subset of metamorphosis-depleted planula proteins likely includes candidate proteins involved in metamorphosis initiation.

The aboral pole in cnidarian planulae contains specialized cell types (Ramon-Mateu et al., 2025), which in anthozoans are organized into planula apical organs, rich in sensory-ciliated cells that direct probing behavior and settlement; single-cell atlases and developmental studies in hydrozoans and anthozoans converge on this polarity model (Sinigaglia et al., 2015; Zang and Nakanishi, 2020; Ramon-Mateu et al., 2025). Here, the aboral enrichment in dynein β chain/ciliary motor proteins plus several cytoskeletal-binding proteins (e.g., enriched microtubule binding GO term under Molecular Function) is consistent with expected adaptations for a planula sensory zone that explore surfaces before settlement.

Planula abundant GPCRs (12 proteins) included an aboral localized rhodopsin-like adhesion GPCR (PFX23700.1) and were depleted in settler (expressing only 2 Guanine nucleotide-binding protein G(I)/G(S)/G(O) subunits). Their planula enrichment pattern is in agreement with previous transcriptomic findings of 9 GPCR transcripts in aboral tissue of *Pocillopora acuta* planula (Ramon-Mateu et al., 2025), and of depleted transcript abundance and shifting GPCR (subfamilies) diversity in *Acropora tenuis* larvae induced to metamorphose by a synthetic *Hym* neuropeptide (Ishii et al., 2022). The presence of many GPCRs in the planula proteome suggests that receptor translation and membrane localization are already established in planula, enabling rapid sensory responses when cues are encountered. Receptor availability is then downregulated in the proteome of metamorphosis-committed settler, consistent with the temporal regulation reported before/after a transcriptional Point of No Return (Ishii et al., 2022).

Cytoskeletal and cytoskeleton-binding proteins, such as settler-upregulated actin, myosin-2 essential light-chain, tropomyosin, and myogenic myotrophin-like proteins, and planula-upregulated F-actin capping protein, provide the smooth muscle contractile machinery typical of coral epithelio-muscular cells (Leclère and Röttinger, 2017). These proteins drive body contractility via re-arrangements of myoepithelial filaments, and muscular-hydraulic mechanisms (Leclère and Röttinger, 2017; Stokkermans et al., 2022). So, their differential abundance shown here aligns with expected changes in contractility and tissue mechanics during metamorphosis associated larval body contraction (oral/aboral axis).

Our analysis also shows a developmental sequence of distinct differentially abundant extra-cellular matrix (ECM) components such as co-adhesins, hemicentins and spondin variants. Planula enriched co-adhesins (Figure 2C) are extracellular matrix glycoproteins that might regulate cell-substrate adhesion and antimicrobial activity. Hemicentin-1 and SCO-spondin initially enriched in planula and localized to aboral pole tissue (Figure 3E) were replaced in metamorphosis-committed settler by enrichment in hemicentin1-like and hemicentin-2, spondin-2-like and thrombospondin-related proteins (Figure 2B). These variants may mediate distinct biological processes by acting at aboral tissue sites and triggering different biochemical responses in cells depending on development stage. Hemicentins are conserved extracellular members of the immunoglobulin superfamily, which organize epithelial and other cell attachments into oriented, line-shaped intercellular junctions (Vogel and Hedgecock, 2001). In adult coral colonies, hemicentin and collagen are ECM components involved in tissue organization and attachment (Hemond et al., 2014), consistent with roles in adhesion and tissue remodelling during settlement. Thrombospondin-type and TSR containing proteins are prominent mesoglea/ECM components in cnidarians and have been linked to ECM organization, cell-ECM interactions and developmental signalling in *Hydra*, e.g., Wnt-regulated expression (Lommel et al., 2018). Transcripts with thrombospondin and von Willebrand A (vWA) domains were reported among genes differentially expressed during coral metamorphosis and settlement (Hayward et al., 2011), linking TSR/vWA-containing ECM proteins to attachment/ECM remodelling during metamorphosis. Transcriptional changes in skeletal/ECM matrix components (including thrombospondin) were also reported during larval settlement in *Pocillopora damicornis* (Mass et al., 2016). Additionally, several co-adhesins and (hemicentin-1, thrombospondin and spondin-2) ECM proteins found here differentially abundant in settler versus planula tissue, have previously been recorded in the skeletal organic matrix proteomes of related *Stylophora* corals (Drake et al., 2013; Mummadisetti et al., 2021), suggesting additional putative functions of these proteins in biocalcification, beyond adhesion. During the metamorphosis transition, these ECM components may contribute to organize a polarized adhesive interface at the aboral pole of the settling larva, which further calcifies into the basal plate, which is the first skeletal element of the forming primary polyp, already visible in settlers.

Interestingly, the detected co-adhesins and hemicentins share similar sequence domain architecture to cnidarian polydom molecules (*CnPolydom)* (Schwarz et al., 2008) with typical multiple domains (Supplementary Table S2) and predicted functions in adhesion, GPCR-sensory signaling (domain IPR000276:GPCR_Rhodpsn) and immunity. This shared structural motif, called the pentraxin domain, classified them to the pentraxin family of immune-related molecules, which are recognition and effector molecules in the innate immune responses of both vertebrates and invertebrates (Schwarz et al., 2008; Mantovani et al., 2008). Upregulation of pentraxin proteins during coral metamorphosis may thus support multiple functions in ECM re-modelling, biocalcification and immunity.

Together, our observations support and extend the previous hypothesis that cytoskeletal re-organization, ECM remodeling and adhesion signaling are coordinately involved during coral larval settlement and metamorphosis (Hayward et al., 2011). Our proteome data supports a role of the aboral pole as a coordinated pre-settlement pole, with neuro-sensory ciliated cells that explore the micro-environment and receive and integrate external cues, activating GPCR signaling pathways, body contraction and shifting ECM composition.

### Morphogenesis and biomineralization related processes

Our proteomics results highlight upregulation of Notch protein at onset of metamorphosis. Transcriptomic studies have indicated that coral larval metamorphosis and settlement activate genes associated to cellular morphogenesis and tissue differentiation (Helman et al., 2008; Reyes-Bermudez et al., 2009; Grasso et al., 2011; Siboni et al., 2014). Notch signaling proteins, e.g., neurogenic locus Notch homolog protein 1-like isoform X1, essential for regulating cell differentiation and tissue patterning, have been implicated in developmental transitions in cnidarians. Notch pathway signaling has indeed been shown to regulate neurogenesis and budding in anthozoan *Nematostella vectensis* (Marlow et al., 2012), while its specific role during coral metamorphosis was not explored. Our findings suggest its likely role in coral metamorphosis regulation.

We found several differentially abundant proteins previously identified as components of the coral biocalcification toolkits (Drake et al., 2013), namely three coral acid rich proteins (CARP1, CARP2, CARP3) and one carbonic anhydrase. The abundance changes of these proteins during metamorphosis is consistent with previous, targeted proteomic and transcriptomic results obtained in the related coral *Stylophora pistillata* (Mass et al., 2013; Akiva et al., 2018; Peled et al., 2020). In particular, CARP2, rich in glutamic acid, has been shown to interact with amorphous calcium carbonate (ACC) nanogranules in planula larvae, suggesting a potential role in precursor stabilization in the early phases of mineral deposition (Akiva et al., 2018). Meanwhile, CARP1, CARP3, and carbonic anhydrase detected in association with aragonite in settling larvae, may contribute to aragonite crystal maturation during post-settlement biomineralization (Akiva et al., 2018). Here, the enrichment of CARP1 in planula stage supports the gene expression patterns reported in *Acropora millepora* larvae (Reyes-Bermúdez and Rodríguez-Cabal, 2024). The presence of multiple EF-hand domains in CARP1 further supports a putative role in calcium sensing at initiation of biocalcification.

### Small metabolites and energy metabolism, proteolytic processes

Planula aboral tissue was enriched in metabolism proteins and domains that support extracellular chemical signal processing, such as peptidases and ATP-dependent small metabolite GHMP kinases, that catalyze amino-acid or isoprene biosynthesis, and galactose metabolism (Bork et al., 1993). Sulfatase and SEA (extracellular) domains were also abundant that may be adjacent to transmembrane glycoproteins and have carbohydrate regulated proteolytic activity. Other small-molecule processing enzymes include TauD domain proteins (taurine catabolism), found in both stages and only in aboral half in planula stage, supporting previous transcriptional findings of TauD enrichment in cells located around the aboral pole in planulae of corals as well as hydrozoans (Ramon-Mateu et al., 2025). Aboral co-enrichment of these enzymes suggests specialized metabolism at the planula sensory/attachment pole, possibly regulating the local microenvironment or modulating chemosensory responses that control settlement.

Other aboral specializations include coordinated CYP450-dependent metabolic activities as shown by colocalization of aboral-enriched steroidogenic cytochrome P450 17 dehydrogenase (CYP17), aminolevulinic acid dehydratase (ALAD, that catalyzes the biosynthesis of porphobilinogen precursor of heme and tetrapyrroles), and arylsulfatase B (a sulfotransferase involved in steroid turnover). At settler stage, a CYP450 3A subfamily protein implicated in cellular xenobiotic detoxification and steroid degradation was found to be significantly enriched. Further studies are needed to detect and quantify levels of steroidal hormones and corticoids in planula and settling larva. In adult *Pocillopora damicornis* tissue, detectable levels of 17β-estradiol, estrone, progesterone and testosterone were reported over two consecutive lunar reproductive cycles, along with activities of the steroidogenic enzymes 3β-hydroxysteroid dehydrogenase and cytochrome P450 17 dehydrogenase (Rougée et al., 2015). It would be interesting to investigate a possible involvement of putative aboral corticosteroids (“stress hormones”) in the regulation of coral planula metamorphosis, in agreement with known corticosteroid modulation of amphibian larval metamorphosis.

A Prostaglandin E synthase2 C-terminal domain (IPR034335) was detected among the 98 planula aboral signature proteins, congruent with the significant planula enrichment of allen oxide-synthase/lipoxygenase (dual function) and alpha-dioxygenase-2 enzymes (heatmap of planula enriched DEPs, abundance levels detailed in Supplementary Table S2). All together these results provide evidence of active LOX/COX pathways (Lõhelaid et al., 2015) in planula, and suggest putative aboral production of pro-inflammatory molecules.

In settlers, several enriched proteins were related to lipid metabolism (e.g., 1-acyl-sn-glycerol-3-phosphate acyltransferase (AGPAT), pancreatic triacylglycerol lipase, enoyl-CoA delta isomerase 1, mitochondrial-like), but also oxidative metabolism (cytochrome P450s, retinol dehydrogenase 8-like) (Fuchs et al., 2014; Pankov et al., 2021), and regulation of membrane endosomal trafficking (RAB11-binding protein, Upstream Stimulatory (transcription) Factor) (Chen et al., 2005; Voss et al., 2023; Chatin et al., 2026). These pathways are consistent with the energetic and metabolic changes that support coral metamorphosis and calcification initiation. Metamorphosis and post-settlement stages are known to be energy demanding (measured as increased respiration and metabolic cost at settlement) (Edmunds et al., 2013), and enrichment in both lipid enzymes and mitochondrial/respiratory proteins are expected signatures of metamorphosis. Indeed, larval and post-settlement studies in scleractinian corals have shown that stored planula lipids are remobilized during competence and settlement, with enzymes that synthesize, hydrolyze or route glycerolipids (AGPATs, lipases, enoyl-CoA isomerases) involved in metabolism during larval dispersal and settlement (Harii et al., 2007; Strader et al., 2018; Randall et al., 2024). Additionally, intracellular endosomal trafficking via RAB11-binding protein has recently been associated to the coral calcification process (Chatin et al., 2026).

A two-phase proteolytic model of coral metamorphosis is proposed based on the stage-specific abundance pattern of metalloproteinase and transport proteins, in agreement with gene expression records in metamorphosing *Acropora* larvae (Ishii et al., 2022). In planula, abundance of membrane-associated proteases (ADAM/ADAMTS family), zinc-metalloproteinases and peptide-processing enzymes is consistent with a pre-metamorphosis phase in which proteolytic machinery is positioned to modulate cell-matrix adhesion, cleave or activate peptide ligands, locally tune receptor availability and prepare extracellular interfaces (functions that maintain the tissue architecture). In settler, the commitment to metamorphosis is characterized by enriched collagenases and other matrix-degrading enzymes, and of protein modules related to proteolysis and transport, indicative of activated protein degradation and tissue reorganization pathways, consistent with Ishii’s transcriptomic observations that proteolytic processes become more prominent after the Point-of-No-Return (Ishii et al., 2022).

### Antioxidant capacity build-up and innate immunity protein enrichment

Compared to planula the settler stage proteome was characterized by significantly increased abundance of a large repertoire of oxidative stress defense proteins, including catalase-like isoforms, glutathione S-transferases (GSTs) and superoxide dismutase (SOD) domains, peroxidasin-like proteins, selenoproteins, and universal stress proteins. The overexpression of reactive oxygen species (ROS) scavenging enzymes illustrates large redox signaling changes that are usually associated across anthozoans to thermal or hypoxic stress and pollutant exposure response (Sunagawa et al., 2008; da Silva Fonseca et al., 2021; Maznan et al., 2024). Extending these observations to settlement physiological processes, it was recently reported that (*Leptastrea purpurea*) coral metamorphosis is mediated by hydrogen peroxide (H_2_O_2_) release resulting from larval cells photodegradation of a bacterial pigment (cycloprodigiosin), and that exogenous H_2_O_2_ alone induces rapid metamorphosis (Petersen et al., 2023). Our findings in *Pocillopora acuta* metamorphosis of upregulated oxidative stress defense proteins and oxidoreductase activity (GO terms) support the hypothesis that redox signaling and oxidative stress processes may regulate coral metamorphosis. The larval machinery of cellular antioxidant proteins detected here may mitigate ROS production by coral phagocytes (Snyder et al., 2021) after potential opsonization processes (see below).

Finally, shifts in innate immunity related proteins were found in our dataset. Planula were enriched in venoms like proline-rich extensin-like EPR1 neurotoxin (Klompen et al., 2020), Kunitz-type actitoxin (Esquinca et al., 2023)) and soluble pattern-recognition proteins (pentraxin domain-containing hemicentin-1 and co-adhesins; LamG/ConA lectin). Settlers were characterized by an expanding repertoire composed of venom (*A.superbus* venom factor 1), several lectin-like proteins (e.g., rhamnose-binding and ricin-like lectins; pentraxin classified hemicentin variants), anti-retroviral barrier-to-autointegration factor (BAF) (Jamin and Wiebe, 2015)) and two BRICHOS-related predicted antimicrobial peptides (uncharacterized proteins CAH3125467 and XP_058945786.1; Supplementary Figure S3).

In the planula stage, the aboral enriched innate immunity related proteins (Toll Like Receptor adaptor protein MyD88; mucin-19-like lectin–glycan; cysteine-rich small proteins; sushi repeat-containing protein SRPX2-like) can be interpreted as pre-settlement immune toolkit. PDIs and small heat shock proteins secure secretory protein folding and prevent aggregation of cysteine-rich/secreted factors. And finally, the acylase enzyme (Penicillin-V acylase (PVA)/Ntn-hydrolase–type) may filter the local biofilm/quorum environment that either promotes or suppresses settlement (Mason et al., 2024). Lectins are mediators of innate immunity and are implicated in coral substrate recognition during settlement (Grasso et al., 2011; Palmer and Traylor-Knowles, 2012). Other Pattern Recognition Receptors (PRRs) in corals may include pentraxins (Schwarz et al., 2008) that mark target damaged tissue or selected microbiota, in a pro-inflammatory opsonization step that contributes to both microbial recognition and homeostatic modulation (Palmer, 2018). Specialized coral phagocytic cell types have been described in *Pocillopora* adult colony tissue, that generate reactive oxygen species within phagolysosomes (Snyder et al., 2021). During settlement, such immune signaling pathways targeted phagocytosis and associated oxidative burst might facilitate autophagy of larval structures and regulate microbial selection and pathogen protection at larval-environment interfaces. Such roles are individually documented in cnidarian and other marine larval literature (Grasso et al., 2008; Muras et al., 2018; D’Ambra and Lauritano, 2020; Palmer and Traylor-Knowles, 2012), and together form a coherent coordinated model highlighting innate immunity activation in planula settlement.

### Model of larval proteome changes at the onset of coral metamorphosis

**FIGURE 4:**
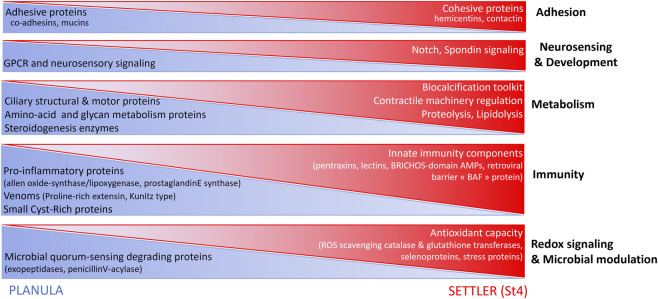
Model of larval physiological changes during coral metamorphosis. A developmental sequence of enriched proteins (with putative functions in adhesion, neuro-sensing and development, metabolism, immunity–although they likely have multiple roles) highlights innate immunity maturation in addition to neuro-muscular and (biocarbonate) skeletal development in settling coral larva. Shifting protein abundances and/or identities are observed between planula/settler stages within each category, supporting downregulation of environmental sensing in metamorphosis-committed larvae, in parallel to tissue remodeling, differentiation of neuronal, myoepithelial and calcified structures, and expansion of immune repertoire. The variable amplitude of larval proteome response to metamorphosis among parent-colony origins is explained by parent-specific developmental speed.

Our results of metamorphosis upregulated protein pathways were integrated with aboral planula protein localization data to highlight larval proteome changes that can be structured as illustrated in (Figure 4).

Major protein categories activated in early metamorphosis include cytoskeleton and ECM adhesion components (from adhesive to cohesive proteins), GPCR neuro-sensing and morphogenesis (Notch and spondin) signaling proteins. Additionally, marked upregulation of oxidative stress defense and innate immunity proteins emerges as an early feature of the metamorphosis transition.

We propose that in planula, ECM remodeling/morphogenesis/calcium-binding skeletal organic matrix proteins, and innate immunity proteins (venoms, pentraxins) prepare an adhesive basal interface supporting metamorphosis and calcification once the optimal combination of biochemical and physical signals is received via G-Protein coupled Receptor signaling. In settlers, the observed build-up of antioxidant defense capacity (ROS scavenging enzymes and proteins) may play multiple roles, including buffering of potential opsonization and phagocytic processes (non-specific innate immune response). Additionally, an expanding repertoire of immune-related lectins and two novel, BRICHOS domain antimicrobial peptides, support innate immune system maturation in the forming coral polyp.

## Conclusion

This work provides a detailed description of changes in coral larval proteomes over the metamorphosis transition. LF-Quantification revealed 102 differentially abundant proteins in settling (stage 4) vs. swimming planula larvae of *Pocillopora acuta* across 4 distinct parent colony origins, of which 96 were annotated and clustered into 7 putative broad functional categories. The coral larval metamorphosis process involves significant enrichment of proteins associated with innate immunity, oxidative stress defense and neuro-sensing, as well as proteins expected to be found in settling, calcifying polyps (related to adhesion and cytoskeleton remodeling, metabolism, morphogenesis and biocalcification). Proteomic variability related to parent colony origin was identified, representing a maternal carry over effect that may modulate the speed of the development sequence at onset of metamorphosis. Protein enrichment patterns at the aboral pole of planula larvae support tissue-level aboral specialization, consistent with a concentration in this region of specialized cell types involved in settlement. Active cell signaling, cytoskeletal rearrangements, and adhesion processes in the aboral region likely relate to probing and selection of benthic substrates suitable for metamorphosis. Overall, our results reveal the maturation of immunity and oxidative stress response for putative larva-to-larva and larva-environment communication during coral settlement.

## Data Availability

The mass spectrometry proteomics data presented in the study are deposited in the PRIDE partner repository of the ProteomeXchange Consortium, with the dataset identifier PXD071713. (M&M l419-422). Detailed protein lists are provided in Supplementary Material tables.
