# Supplementary figures and images for: Coral larval proteomics at onset of metamorphosis highlights innate immunity maturation in parallel to neuro-sensing and skeletal development
Source: Front Physiol. 2026 Feb 19;17:1763453. doi: 10.3389/fphys.2026.1763453 (PMC12960093; doi:10.3389/fphys.2026.1763453)

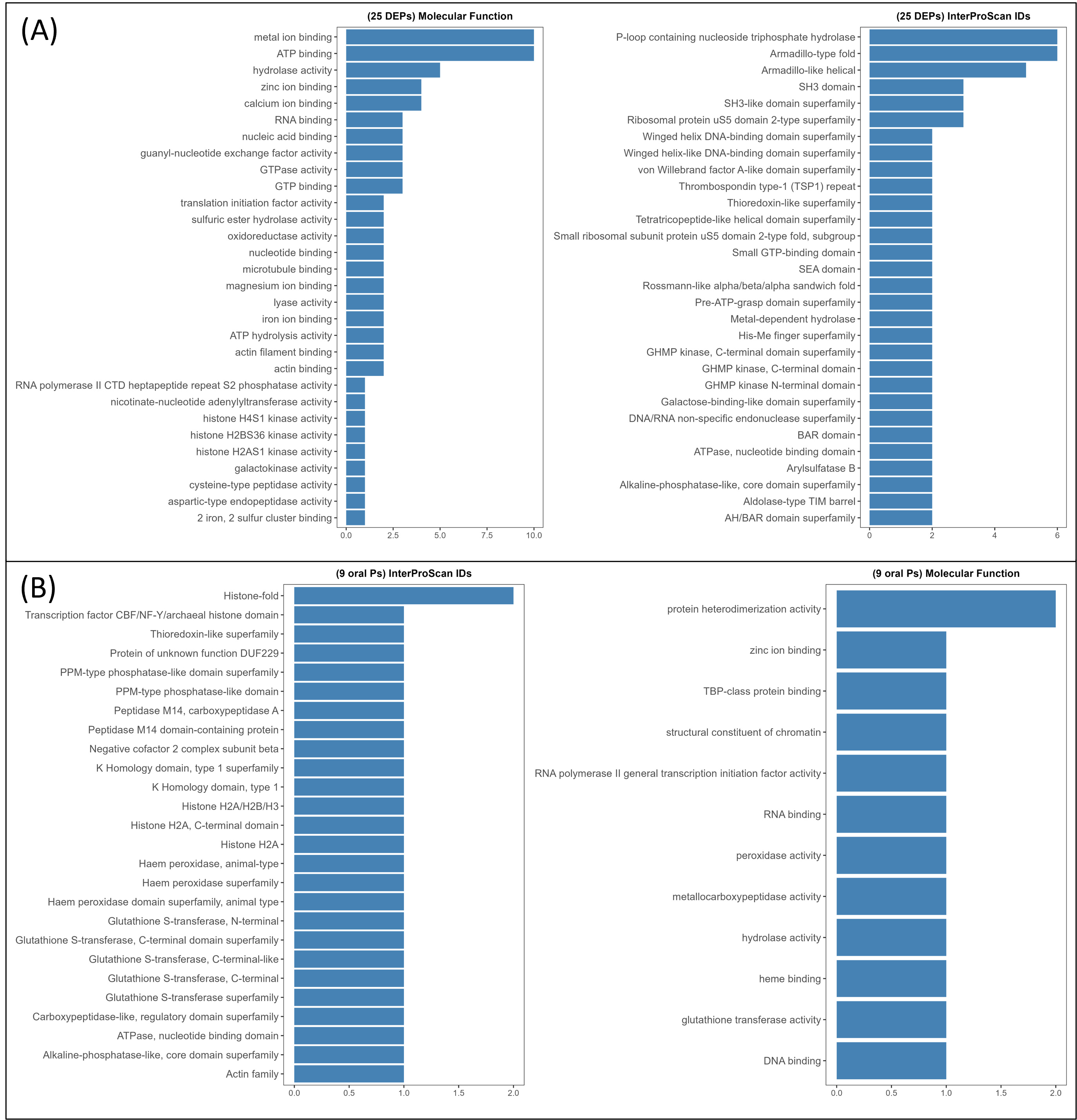

Supplement: Supplementary file 3 [file Image6.tif]

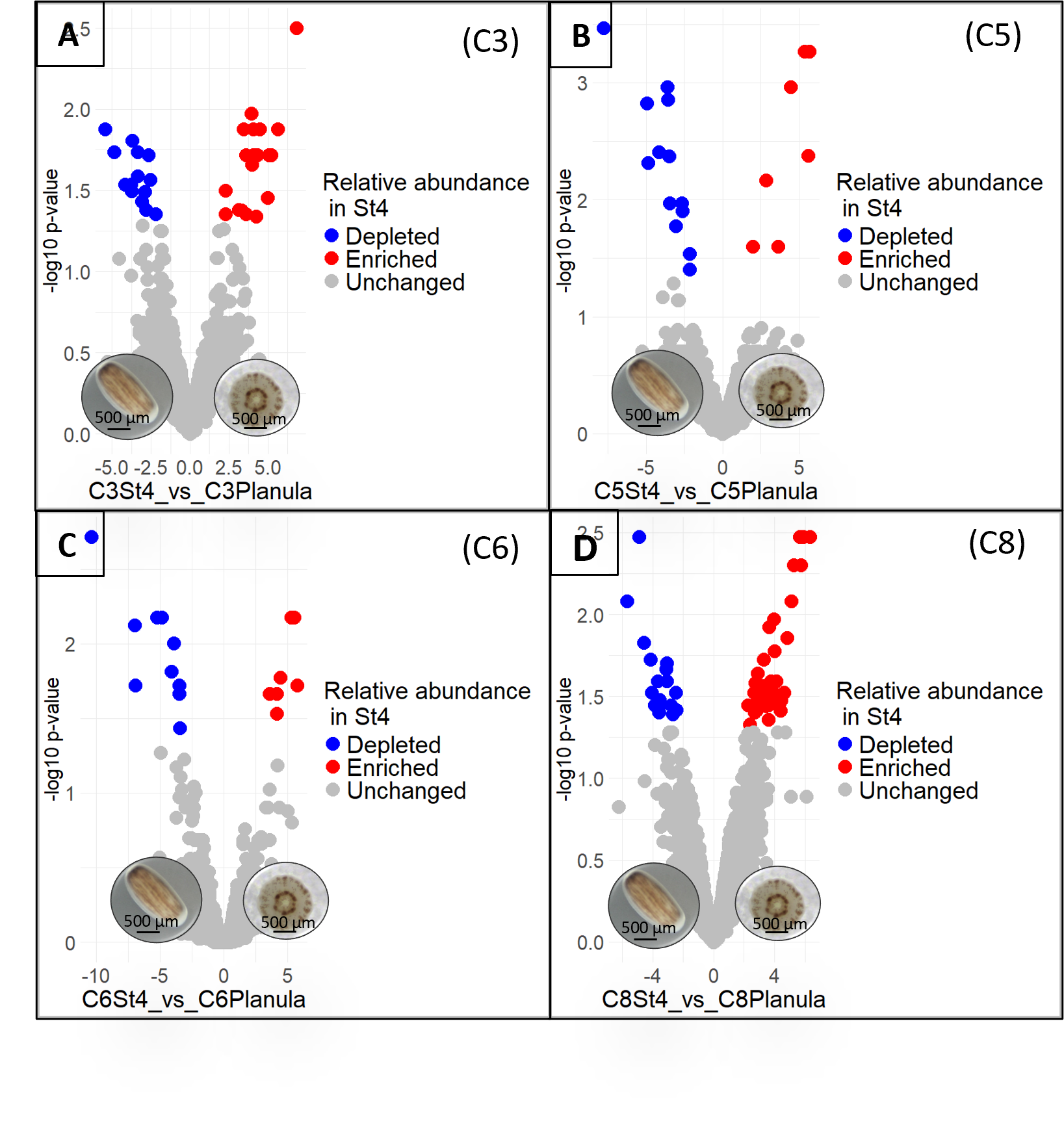

Supplement: Supplementary file 5 [file Image3.tif]

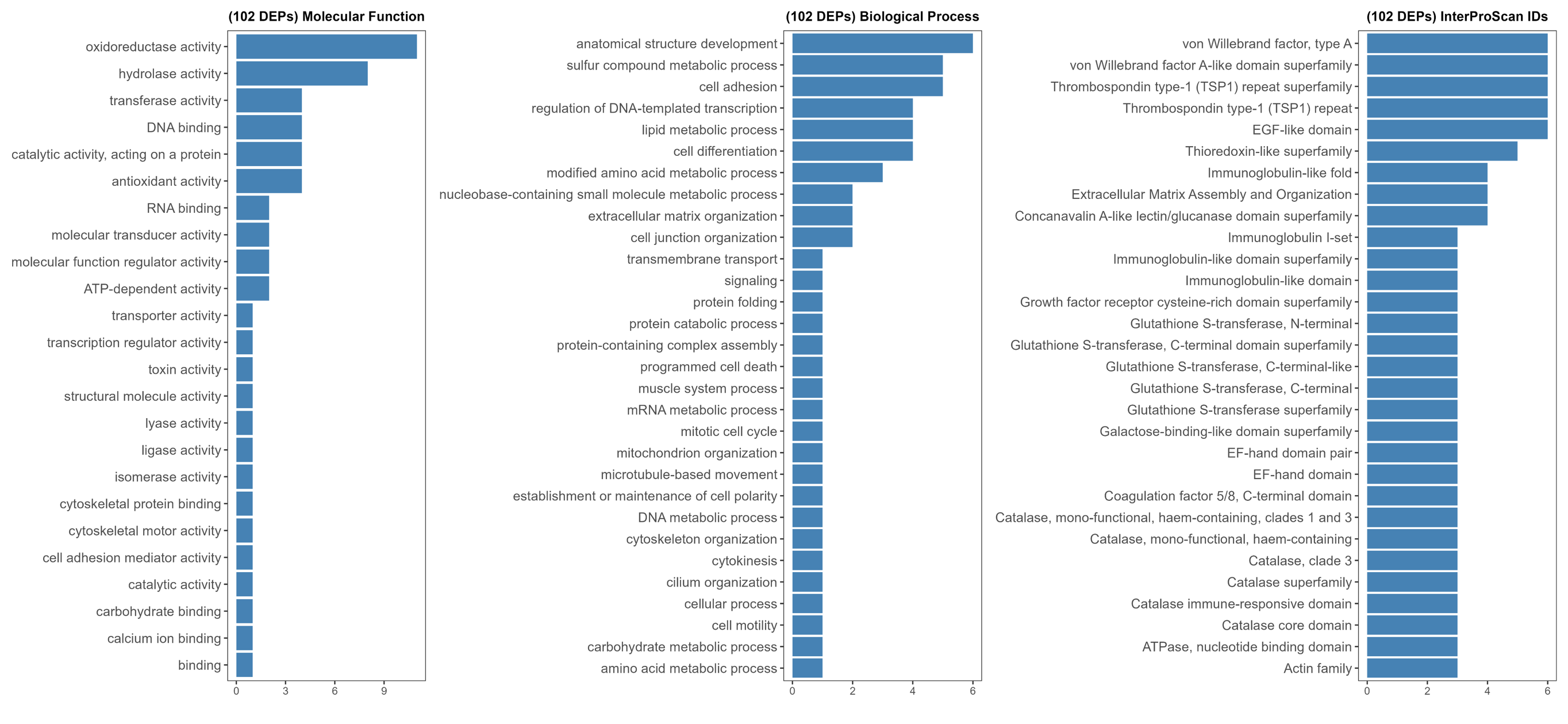

Supplement: Supplementary file 6 [file Image4.tif]

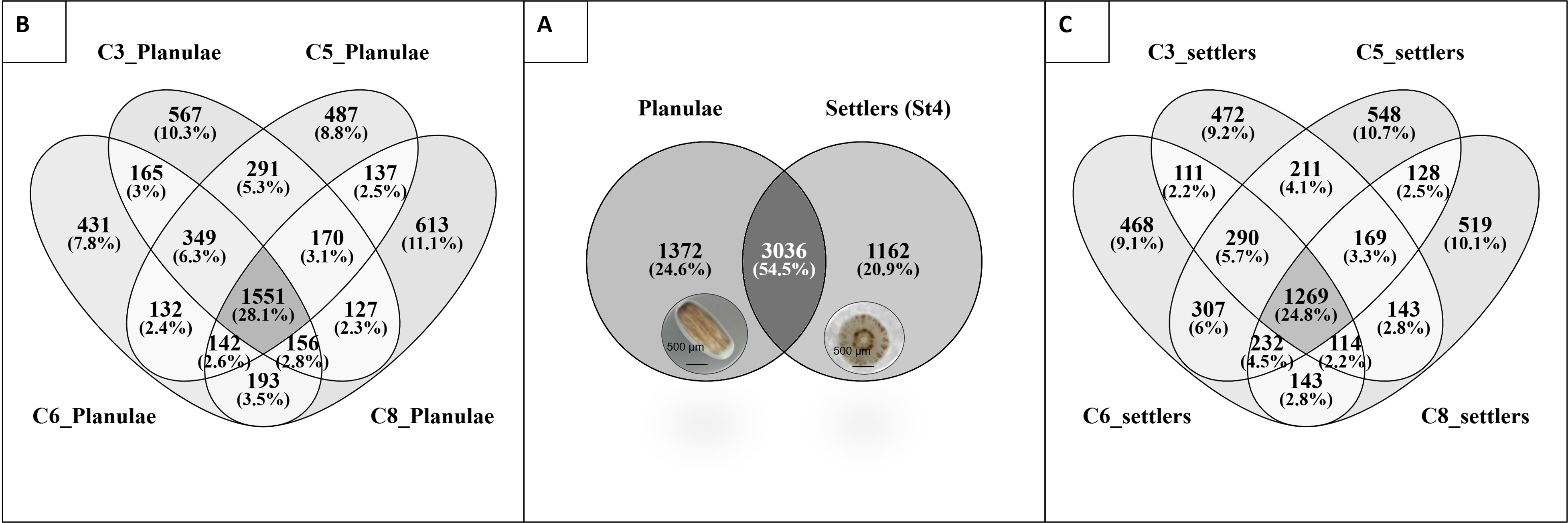

Supplement: Supplementary file 7 [file Image2.tif]

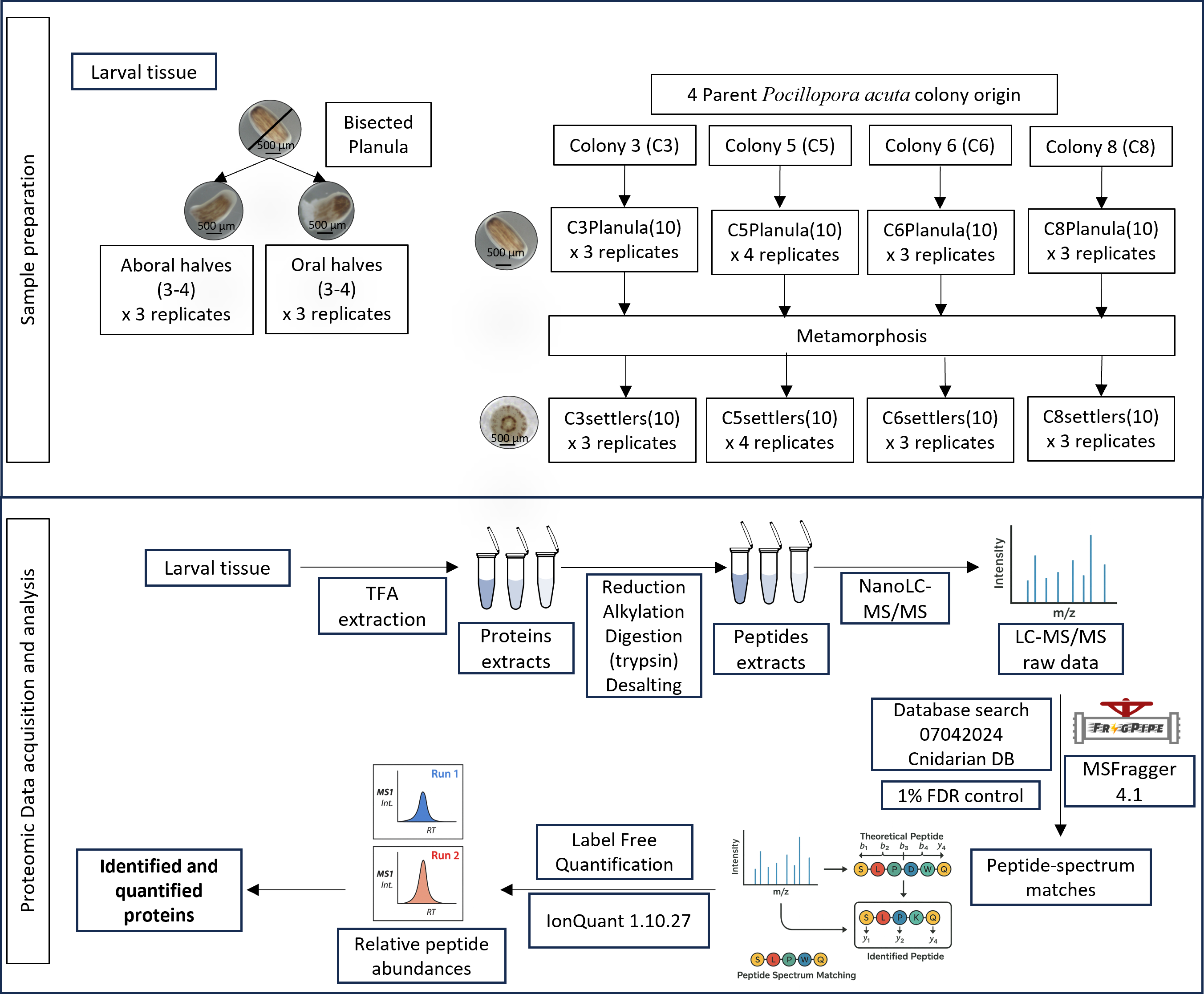

Supplement: Supplementary file 8 [file Image1.tif]

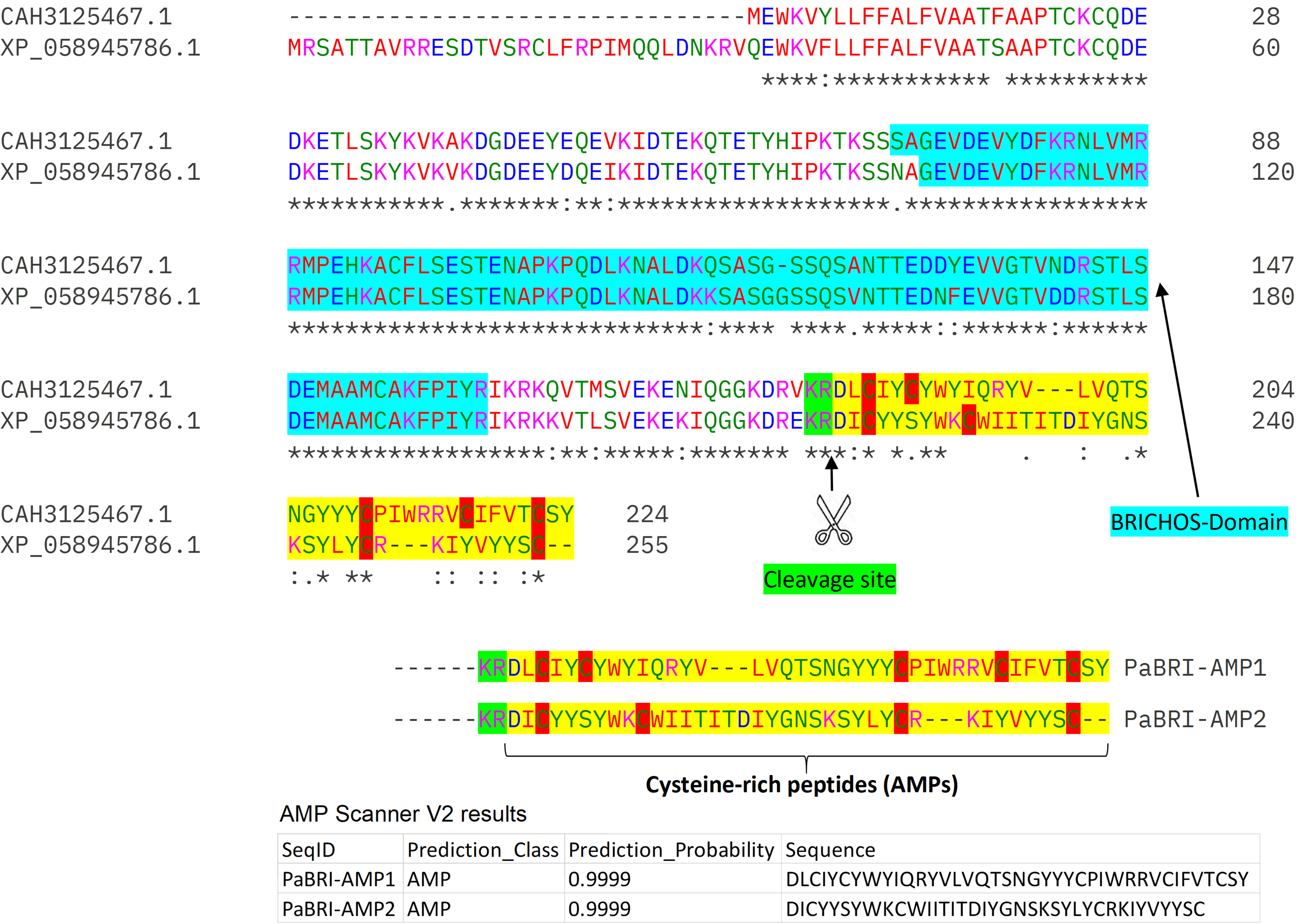

Supplement: Supplementary file 11 [file Image5.tif]
