# Supplementary material for: Coral larval proteomics at onset of metamorphosis highlights innate immunity maturation in parallel to neuro-sensing and skeletal development
Source: Front Physiol. 2026 Feb 19;17:1763453. doi: 10.3389/fphys.2026.1763453 (PMC12960093; doi:10.3389/fphys.2026.1763453)
Supplement: Supplementary file 4 [file Supplementaryfile1.docx]

**Supplementary material captions:**

**Supplementary Fig. 1 Sampling design and proteomic workflow.** Planula larvae released by 4 distinct parent colonies spontaneously initiated metamorphosis, reaching settler (st4) stage. Proteins were extracted from *Pocillopora acuta* Lamarck 1816 larvae using trifluoroacetic acid (TFA), then digested into peptides with trypsin. The resulting peptides were separated by liquid chromatography, sprayed into a mass spectrometer which measures the ionized peptides mass-to-charge values (m/z) and charges. Each of a selected set of peptides were then separately isolated and fragmented inside the mass spectrometer, and the fragments were measured. The resulting raw data were analyzed using FragPipe V 22.0 to identify the amino acid sequences of the peptides and measure their relative abundances, which are used to infer the protein identities and relative abundances.

**Supplementary Table S1.** **Summary of NanoLC–MS proteome analyses of coral larval tissue.**
Synthetic overview of proteomic features of coral larval extracts. Coral proteins were identified using either Peaks (1,600–2,300 protein groups) or FragPipe (1,900–3,100 proteins) algorithms, based on the *Cnidaria07042024* protein sequence database. The total number of identified proteins was consistently higher with FragPipe, which workflow was therefore selected for further proteome detection (bisected planulae) and all label-free quantification (LFQ) analyses (bisected planulae and whole planulae / settlers). A significantly higher number of proteins was detected in aboral compared with oral half-planulae (*p* < 0.05). In addition, proteins of **Symbiodiniaceae origin** were also identified and quantified using the FragPipe analytical pipeline and a *Symbiodinaceae16052025* protein sequence database.

**Supplementary Fig. 2** **Unique and shared *Pocillopora acuta* larval proteins*.*** (A) detected across both development stages and independently of parent colony origin (total 2x13 = 26 samples, FragPipe protein detection workflow): 54.5% of proteins are shared with only 24.6% - 20.9% proteins specific to each stage; (B) detected in planulae of 4 parent colonies (C3, C5, C6 or C8) (total n = 13 samples): despite high inter-parent colony variability, about 30% of planula proteins are shared; (C) detected in settlers (St4) of 4 parent colonies (total n = 13 samples): despite high inter-parent colony variability, about 25% of settlers’ proteins are shared.

**Supplementary Fig. 3** **Differentially enriched coral proteins (DEPs) in Settling larvae (St4) vs Planulae from 4 distinct parent colonies.** (A, B, C, D) Volcano plots of log2 fold-change ratio vs log10 p-value show differentially enriched proteins (DEPs) in larvae from colonies C3 (n= 2x3 samples), C5 (n= 2x4 samples), C6 (n= 2x3 samples), C8 respectively (n= 2x3 samples). Fold change > |2| and p < 0.05 were set as the threshold for significant differential abundance.

**Supplementary Table S2. Detailed annotations of differentially enriched proteins (DEPs) identified by LFQ proteomics (FragPipe) of larval samples.** The descriptions, annotations, and fold change ratios of the DEPs identified in comparative analyses of *Pocillopora acuta* proteomes are listed in the following Excel file. **Sheet 1 – larval_DEPs-2stages:** List of 102 coral proteins differentially enriched in settler “stage 4” (58 upregulated proteins) or planula (44 upregulated proteins). Columns include log₂ expression ratios (St4 vs. Pl) in each parent colony origin (C3, C5, C6, C8) – significant fold changes (p_adj < 0.05 and FC > |2|) obtained in least one parent colony origin are highlighted in bold, completed by non-significant fold change values measured in larvae of other parent colonies -, our protein code, RefSeq ID (from BLASTP results), E-value, protein description (BLASTP best match and organism), InterPro domains, putative (literature-predicted) functional category, and references. **Sheet 2 – planula_aboralDEPs:** List of 25 coral proteins upregulated in the aboral half of planula-stage larvae, with the same annotation and quantitative information as in Sheet 1. **Sheet 3 – Symbiodiniaceae_DEPs-2stages):** List of 6 Symbiodiniaceae proteins differentially enriched in planula and stage 4 larvae, including 4 upregulated in planulae and 2 in settlers.

**Supplementary Table S3** Detailed annotations of 1,119 development stage specific, quantified proteins showing differential presence/absence between planula (827) and stage 4 (329), filtered by stage.

**Supplementary Table S4** Detailed annotations of 98 tissue-level, aboral specific, quantified planula proteins showing differential presence/absence between aboral *vs* oral halves.

**Supplementary Fig. 4** Gene Ontology [Molecular Function] terms and InterProScan domain, repeat, and family distribution associated with the 102 Differentially Enriched Proteins in settler *vs* planula stage (6 DEPs were not annotated).

**Supplementary Fig. 5 Sequence alignment of two BRICHOS-related antimicrobial peptides specific to metamorphosing *Pocillopora acuta* (st4) larvae**. Aligned amino acid sequences of CAH3125467.1 and XP_058945786.1 unknown proteins detected only in stage 4 (settlers) feature the conserved BRICHOS domain core structure (highlighted in blue; trypsic peptides match (4/9 and 1/5 respectively) outside of the conserved BRICHOS sequence). Potential furin-like protease processing sites (marked with scissors) are positioned immediately after the BRICHOS domain, following the conserved KR (Lys-Arg) cleavage site. Proteolytic cleavage at these sites would generate mature cysteine-rich antimicrobial peptides (putative PaBRI-AMP1 and PaBRI-AMP2, highlighted in yellow). Cysteine residues (highlighted in red) suggest 2 disulfide bonds per potential β-hairpin peptide. Antimicrobial peptide (AMP) activity is predicted with probability > 0.999 with Antimicrobial Peptide Scanner vr.2 (Veltri et al., 2018).

**Supplementary Fig. 6** Gene Ontology [Molecular Function] terms and InterProScan domain, repeat, and family distribution associated with the 25 aboral Differentially Expressed planula Proteins (A) and the 9 oral planula Proteins (B) (unique to oral pole tissue) in bisected larvae.
